# Impact of temperature and humidity on the structural and biocompatibility of 3D-Printed PLA scaffolds for bone regeneration

**DOI:** 10.1038/s41598-026-51548-1

**Published:** 2026-05-18

**Authors:** Rachelle Gomez-Guevara, Vivek Kamat, Basil Usama Hamed, Juan Pretell-Mazzini, Shekhar Bhansali, Anamika Prasad

**Affiliations:** 1 Department of Biomedical Engineering, Florida International University, Miami, FL USA; 2 Department of Electrical and Computer Engineering, Vanderbilt University, Nashville, TN USA; 3 Baptist Health of South Florida, Musculoskeletal Oncology Division, Miami Cancer Institute, Miami, FL USA; 4 Department of Mechanical and Materials Engineering, Florida International University, Miami, FL USA

**Keywords:** 3D printing, Tissue engineering, Bone Scaffold, Fused deposition modeling, Biocompatibility, In vitro toxicity, Biotechnology, Engineering, Materials science

## Abstract

Fused deposition modeling (FDM) is widely used in medical applications and provides a promising, cost-effective, and user-friendly solution to point-of-care environments. However, this on-site production necessitates extreme process reproducibility in ambient conditions. Although such a requirement is necessary, the effects of environmental temperature and relative humidity during fabrication are poorly understood, especially when complex porous structures are considered. In this paper, we systematically investigate the impact of ambient temperature and relative humidity on the structural, mechanical, and biological performance of porous polylactic acid (PLA) scaffolds fabricated via FDM. Cylindrical porous scaffolds (2.5 cm diameter and heights of 5.3 and 10.3 cm) were printed under controlled conditions in ambient temperature (25–40 °C) and relative humidity (30–70%). Their pore structure (size and density), water-holding capacity, compressive hardness, and in-vitro cytocompatibility were investigated. The geometric fidelity and pore morphology of all scaffolds were similar across fabrication conditions, suggesting that ambient conditions did not influence the qualities in macroscopic visual prints. In comparison, the compressive Young’s modulus increased with increasing temperature. A biocompatibility assay showed that variations in relative humidity had minimal effects on the mechanical performance of the scaffolds but affected cell viability and reactive oxygen species (ROS) generation. Conversely, at higher fabrication temperatures, high intracellular ROS activity was observed without affecting the structural integrity of the scaffolds. These findings establish a practical processing window of moderate temperatures (25–35 °C) and low-to-moderate humidity (30–50% RH) that balances mechanical stability with biological compatibility. These insights into environmental dependence can be used to enhance process reliability and repeatability, which are essential for translating scaffolds printed by FDM into low-cost, high-fidelity clinical and point-of-care applications.

## Introduction

3D printing has revolutionized the health care industry by enabling unprecedented levels of customization and facilitating on-demand production, thereby reducing the need for large inventory stocks^1,2^. Fused deposition modeling (FDM), a type of additive manufacturing technique based on the layer-by-layer extrusion of thermoplastic filaments, has become one of the most adopted methods in both engineering and biomedical settings. In 2023, the global FDM market is estimated to be worth USD 1.7 billion and is expected to grow at a 21.8% compound annual growth rate, indicating its wide-ranging accessibility and maturity of the technology^3,4^. FDM offers a practical and scalable choice for clinical applications owing to the cost-effectiveness of devices and filaments, ease of use, versatility with design and filament types, and compatibility with diverse biocompatible filaments. Its simplicity eliminates the need for highly specialized technicians, allowing for a rapid expansion of the technology from engineering prototypes to clinical settings, including in resource-limited environments worldwide. Hence, FDM has quickly permeated the medical field, such as anatomical models^5,6^, customized prosthetics^7,8^, and personalized implants and scaffolds^9,10^. A major part of this exponential rise is attributed to the broad selection of commercially available filaments, their distinct material properties, and their compatibility across FDM platforms. Polylactic acid (PLA) is emerging as a predominant filament choice for FDM due to its affordability, ease of processing, low shrinkage, biocompatibility, low cytotoxicity, and favorable thermomechanical properties^11–14^.

Although FDM 3D printing and the use of PLA filament for FDM are both popular choices, several shortcomings remain. FDM innately struggles with quality control when fabricating complex geometries such as highly porous scaffolds, and reliance on support structures for fine prints, which introduces additional challenges in post-processing^12,15,16^. From a material point of view, PLA has lower mechanical strength, limiting its application space, and suffers from lifespan challenges due to its low heat resistance. Both limitations can be further influenced by a wide range of variables, including ambient environmental conditions, filament quality, and printer settings. Collectively, these contribute to print inconsistencies, high print errors, and low reproducibility, particularly when printing fine features required for biomedical scaffolds. Hence, for wide-scale biomedical applications with controlled outcomes, there is a need to quantify errors and optimize process parameters to improve quality consistency.

Several earlier studies have discussed the optimization and effect of process parameters on the quality of FDM prints^15,17^. Parameters such as nozzle temperature, layer height, bed temperature, building orientation, printing speed, screw type, infill density, infill pattern, nozzle diameter, and raster angle are commonly explored^17,18^. Additional studies have attempted to improve the mechanical and functional performance of PLA by incorporating additives such as metal nanoparticles, carbon nanotubes, graphene nanoplatelets, and continuous fibers^12,19,20^. However, ambient factors such as humidity and temperature are often overlooked, despite potentially playing a critical role in the overall printing process and altering material properties, with long-term implications for biomedical applications.

**Fig. 1:**
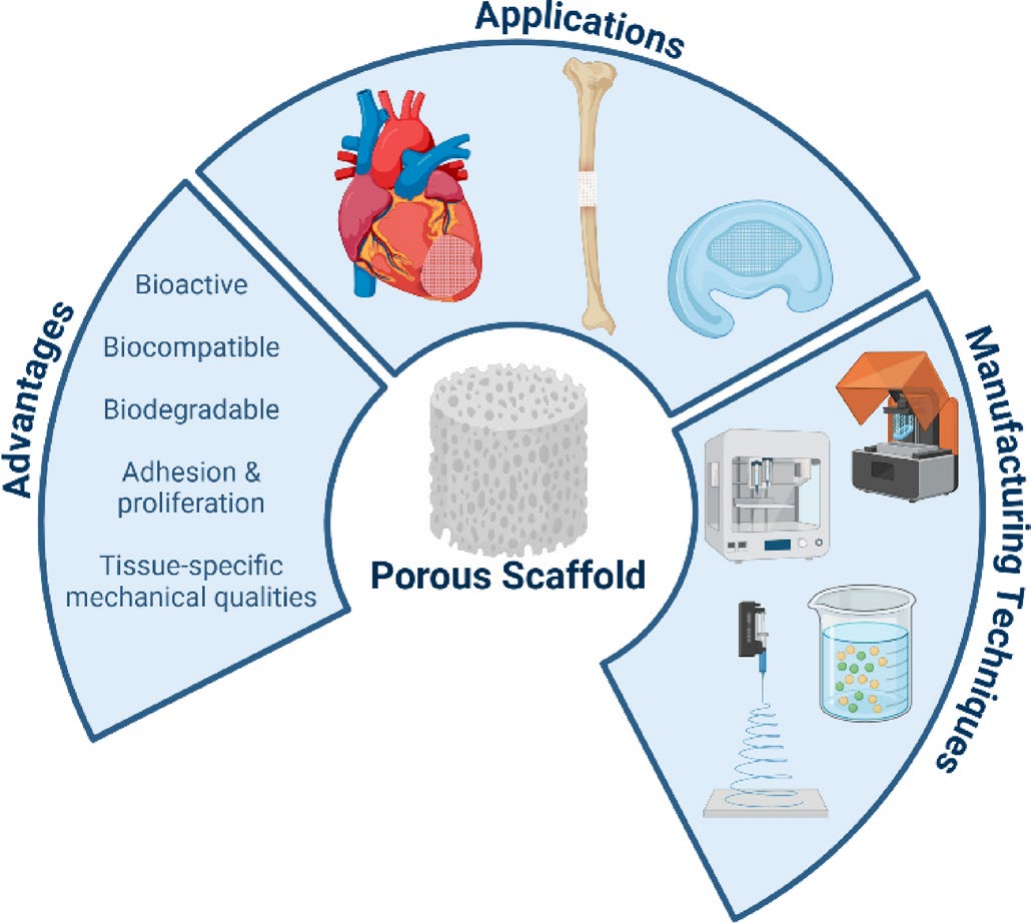
Advantages, applications, and manufacturing techniques of porous scaffolds (image regenerated using BioRender).

Hence, this study systematically investigates the effects of ambient temperature and relative humidity on the print quality and functional performance of FDM-printed polylactic acid (PLA) scaffolds. Porous scaffolds were selected for their complex microstructure and their relevance in tissue regeneration across applications (Fig. 1). Multiple fabrication methods for tissue regeneration are actively being investigated, such as solvent-cast particulate leaching, electrospinning, and stereolithography, each with their distinct advantages^21–24^. Compared to other methods, FDM has the advantage of simplicity, expansive material choices, and the ability to replicate complex computer-aided design (CAD) models, allowing for customization. Especially in bone tissue, they find application in tumor resections, osteomyelitis, infections, nonunions after fractures, and general trauma from injuries^25,26^. In such applications, a porous scaffold is desired over a solid implant to support bone’s inherent regenerative capacity, yet this remains a major clinical challenge. Understanding how environmental temperature and humidity influence print quality enables more controlled, predictable, and error-free scaffold fabrication in clinical or point-of-care settings, ultimately improving production quality, consistency, and therapeutic outcomes. The study will design these scaffolds and print them across a range of ambient temperatures and humidity levels. The print quality will be examined by quantifying changes in pore size, pore density, Young’s modulus under uniaxial compression, water capacity, and in vitro biocompatibility.

## Results and discussion

A PLA porous scaffold was designed in Blender and SolidWorks to mimic the femoral diaphysis; the design was then sliced and printed in eufyMake at two heights, 5.3 cm and 10.3 cm, under variable temperature and humidity conditions representing the ambient office environment. The scaffolds were categorized into room environment (RE), temperature-varied (T25, T30, T35, T40), and humidity-varied (RH30, RH50, RH70) based on the fabrication conditions outlined in the methods section. The fabricated scaffolds were tested for dimensional consistency, pore structure, pore density, water-holding capacity, compressive Young’s modulus, and in vitro biocompatibility.

### Print outcomes under variable temperature and humidity settings

**Fig. 2:**
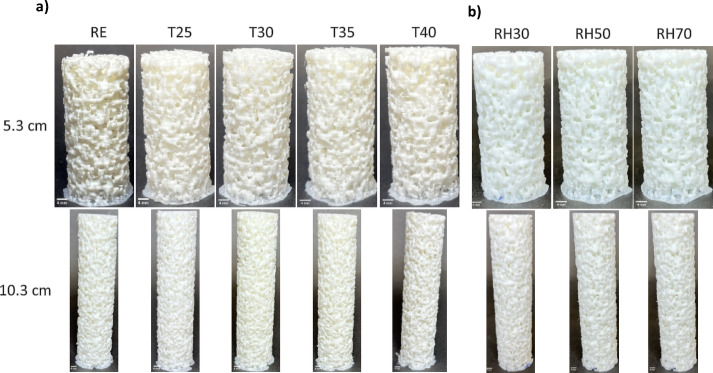
FDM printed PLA scaffolds each with 2.5 cm diameter and two different heights of 5.3 cm and 10.3 cm under temperature and humidity controls **(a)** room environment scaffold printed in room conditions (23.3 ± 0.98 °C, 48% RH) and temperature scaffolds printed at varying temperature levels and constant humidity (48 ± 3%RH), **(b)** humidity scaffolds printed at varying humidity levels, all under constant temperature of 25 °C.

Figure 2 shows representative scaffolds printed at varying temperature and humidity conditions. The 5.3 cm scaffolds were built in 3 h, whilst the 10.3 cm scaffolds were built in about 6 h. No macroscopic failures in prints, including distortion, layer delamination, print breaks, or gross defects, were observed at any temperature or humidity condition. These findings suggest consistent extrusion behavior and homogeneous filament deposition during the ambient conditions tested. High geometric fidelity was demonstrated by dimensional analysis across all scaffolds. The nominal scaffold heights of 5.3 and 10.3 cm produced scaffolds with average scaffold diameters of 24.84 ± 0.067 mm and average heights of 5.31 ± 0.008 cm and 10.31 ± 0.010 cm, respectively. The small standard deviations indicate that temperature and humidity changes in the atmosphere did not significantly disrupt global dimensional accuracy. This dimensional stability concerns point-of-care fabrication, where strict environmental isolation may not be possible.

#### Geometry features

**Fig. 3:**
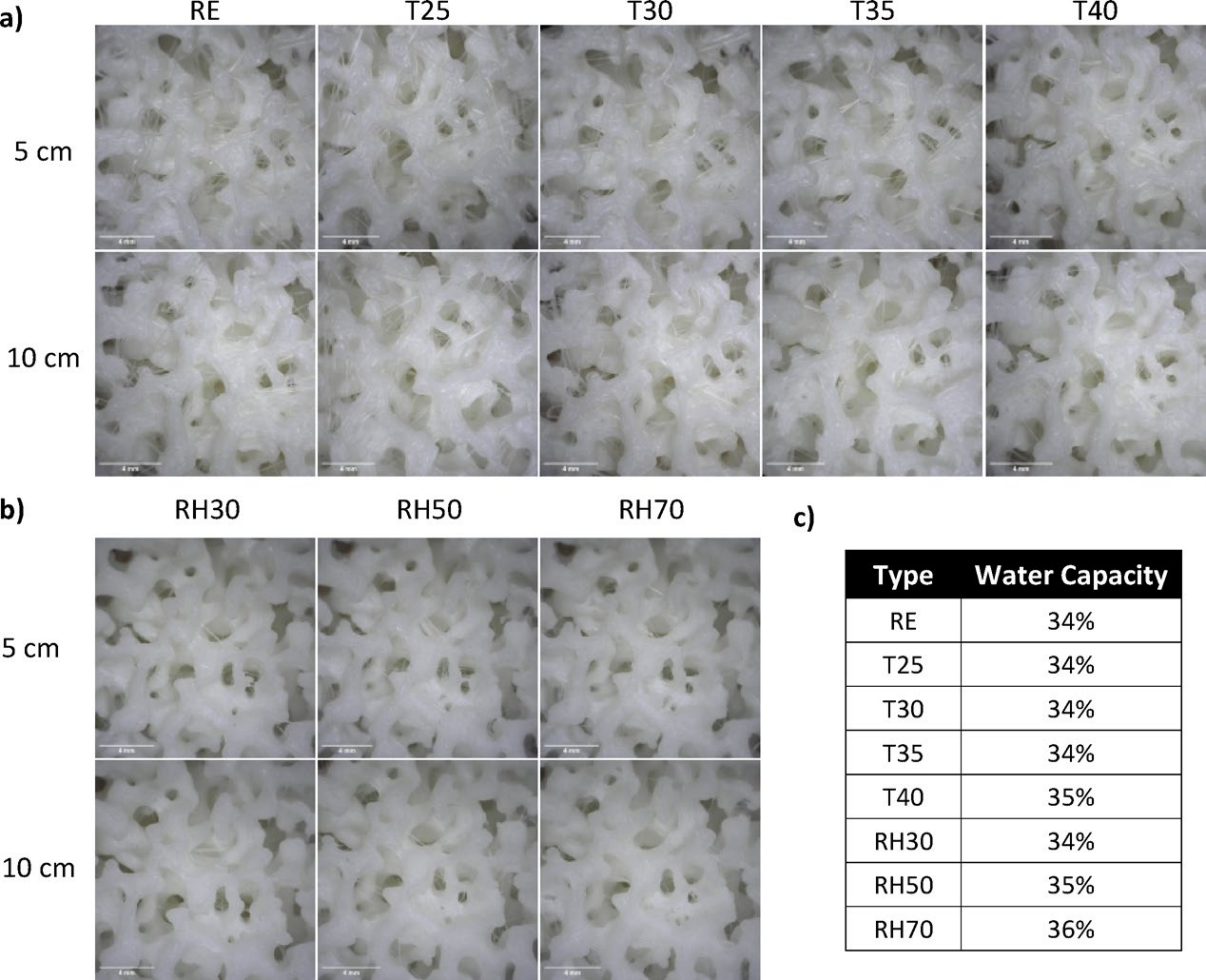
Close-up images of porous scaffold utilized for pore size and pore density calculations **(a)** room environment and temperature variations, **(b)** humidity variations, **(c)** water capacity table.

Figure 3 provides a magnified view of pores for both temperature and humidity levels. The pore size and pore density of each scaffold were determined using ImageJ and a MATLAB script, as described in Sect. 3.3. The average pore size and pore density for the “temperature” scaffolds were 0.64 ± 0.08 mm and 0.15 ± 0.03 pore/mm^2^. The average pore size and pore density for the humidity scaffolds were 0.69 ± 0.06 mm and 0.11 ± 0.03 pore/mm^2^. Overall, the scaffolds had an average pore size of 0.66 ± 0.08 mm and an average pore density of 0.13 ± 0.03 pores/mm^2^. These measurements, with low standard deviation, show consistent printing, which was the primary objective. At the same time, such measurements suffer from inherent limitations, including approximating random shapes as circular and using two-dimensional images.

#### Water capacity

Scaffolds fabricated in controlled-temperature environments had a water capacity of nearly 34%, with an incremental increase of 35% observed in scaffolds printed at 40 °C. The water-holding capacity of scaffolds fabricated at varying humidity increased by 34% for RH30, 35% for RH50, and 36% for RH70. The mean water absorption ability of all the scaffolds was 34.5% ± 0.71%. According to these findings, ambient humidity can have a small effect on the internal pore accessibility or surface wettability, but the observed variation is slight.

Taken together, the dimensional, architectural, and water-absorption analyses suggest that gross print fidelity is not affected by changes in ambient temperature and humidity within the studied ranges. Instead, the effects of the environment are mainly represented by subtle microstructural changes that can lead to downstream long-term mechanical or biological changes.

### Compression testing for young’s modulus

To reduce buckling effects, compressive mechanical tests were conducted on the 5.3 cm scaffolds with the recommended 2:1 length-to-diameter ratio. To account for the reduced load-bearing area in the scaffold due to porosity, the effective contact area was adjusted based on the projected surface area of the STL geometry (177.72 mm^2^), which corresponds to a planar porosity of about 0.36. This was necessary to acquire a representative estimate of the intrinsic compressive stiffness of porous architecture.

**Fig. 4:**
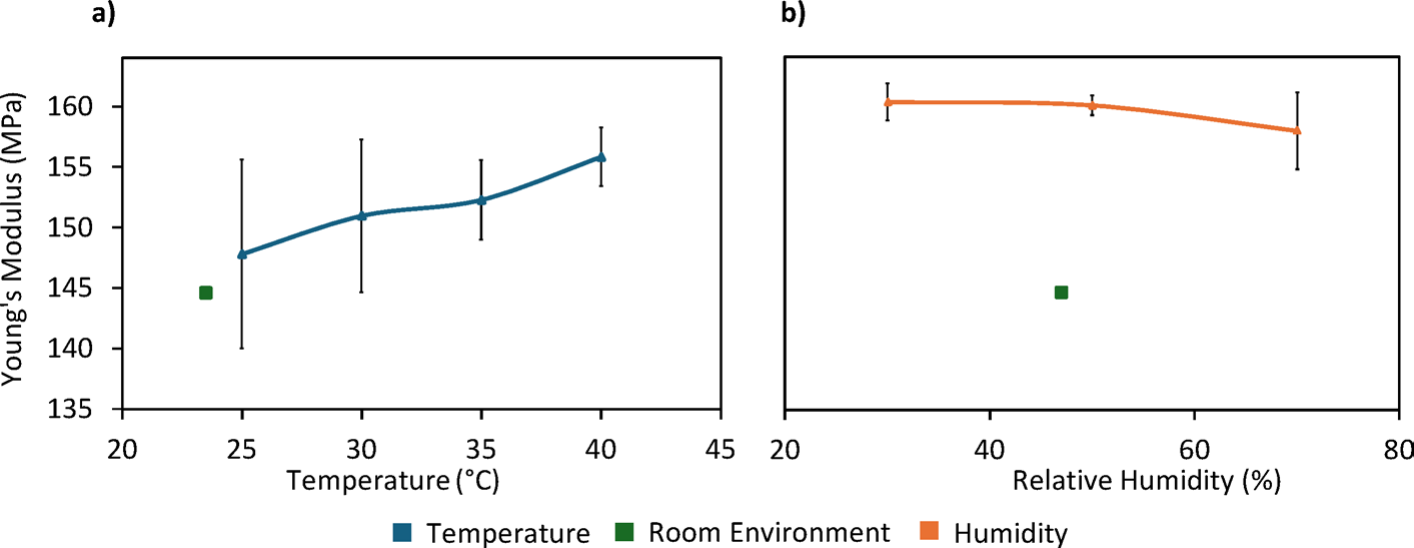
Average compressive Young’s Modulus as **(a)** temperature increases, **(b)** humidity increases, both graphs provide a reference to the average compressive Young’s Modulus of the room environment scaffolds.

The test results are shown in Fig. 4. The average Young’s Modulus of the temperature scaffolds was found to be 144.6 ± 2.8 MPa for RE, 147.80 ± 7.80 MPa for T25, 150.96 ± 6.30 MPa for T30, 152.28 ± 3.27 MPa for T35, and 155.83 ± 2.43 MPa for T40. From these values, it was observed that the Young’s Modulus increased with increasing ambient temperature. The observed phenomenon can be attributed to enhanced interlayer diffusion and bonding due to the high environmental temperatures and minimized thermal gradients during deposition, as well as to facilitated interpenetration of polymer chains at filament interfaces. This increase in Young’s Modulus is also predicted to occur due to reduced voids between print layers, facilitated by easier flow, resulting in reduced stress concentrations and improved bonding between print layers at the increased temperature^27^. Notably, the absolute changes in Young’s modulus across conditions are quite moderate, suggesting that ambient temperature serves more as a tuning parameter than a dominant determinant of mechanical performance in the studied conditions.

Conversely, humidity-varying scaffolds had a relatively constant compressive stiffness at lower humidity, with Young’s moduli of 160.29 ± 1.524 and 160.02 ± 0.799 MPa at RH30 and RH50, respectively. It was reduced to 157.93 ± 3.161 MPa for RH70 with a modest decrease. Higher ambient humidity can add residual moisture to the filament or alter cooling kinetics, thereby enhancing microvoid development or reducing interlayer adhesion. Nevertheless, the extent to which the changes were observed was within experimental variability, indicating that humidity has a secondary effect on compressive stiffness compared with temperature.

### Biocompatibility evaluation of printed scaffolds

**Fig. 5:**
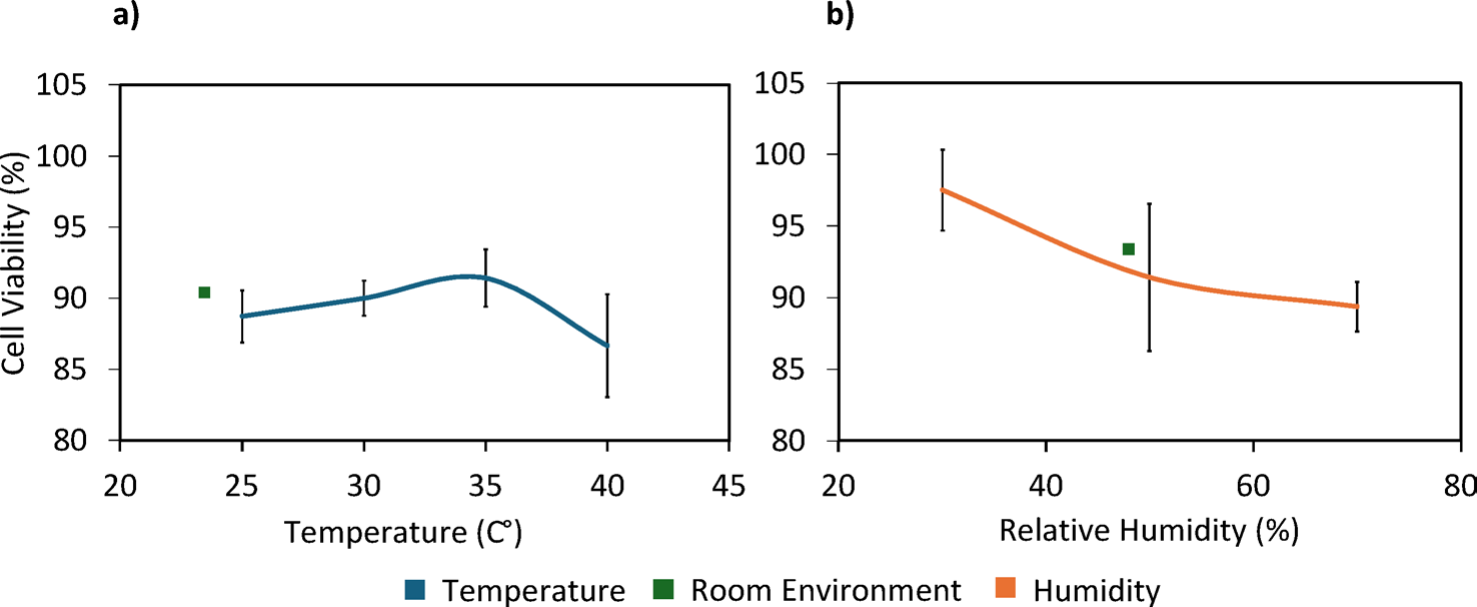
Cell viability outcomes for the **(a)** temperature and **(b)** humidity scaffolds. The viability counts were above 85% across all cases, though the case with the highest temperature (T40) and humidity (RH70) showed a lower value than its counterparts. RH30 showed the highest viability at 97%.

The effect of environmental printing temperature and humidity on cell viability was assessed using an XTT (sodium 3ʹ-[1-(phenylamino)-carbonyl]−3,4-tetrazolium]-bis (4 methoxy-6-nitro) benzene-sulfonic acid hydrate) based metabolic activity assay, which was supplemented with fluorescence-based imaging of the conditions (temperature or humidity showing change in viability) to determine if there is expression of oxidative stress. Figure 5a and b give the quantitative XTT results (based on printing temperature and humidity), respectively.

#### XTT metabolic activity assay for cell viability

Osteoblasts cultured with PLA scaffolds showed high cell viability under all the studied temperature conditions, and XTT -derived viability measures were above 85% (Fig. 5a). Scaffolds prepared at 25, 30, and 35 °C all exhibited steady, high metabolic activity, with values of about 89%−91%, indicating that small changes in ambient printing temperature do not negatively impact scaffold cytocompatibility. Conversely, scaffolds cultured at 40 °C showed a slight decline in XTT signal, with metabolic activity decreasing to around 85–86%. Since the reduction in cell viability was less than 30%, it is not considered cytotoxic (per ISO 10993-5:2009). The results indicate that at high printing temperature, subtle changes in surface or bulk scaffold properties can be induced, which have a minor impact on cellular metabolic performance.

Cellular metabolic activity was highest in scaffolds produced at 30% relative humidity (RH30), reaching about 97%, which was higher than that of the RE group (93%). This increase implies that reduced-humidity environments during printing can promote desirable surface properties, e.g., enhanced polymer consolidation or lower residual moisture, which is better for cellular activity. On the other hand, as humidity levels rose, the XTT signal gradually decreased, with metabolic activity dropping to about 91% at RH50 and about 89% at RH70. These findings suggest that very high ambient humidity levels during fabrication can negatively affect scaffold microstructure or surface chemistry, thereby affecting mitochondrial activity and cellular energy metabolism. Although there are slight deviations, the metabolic activity recognized cytocompatibility of 70–100% was still preserved across all samples, highlighting the natural biocompatibility of the PLA scaffold across a wide range of fabrication conditions.

#### Assessment of reactive oxygen species

**Fig. 6:**
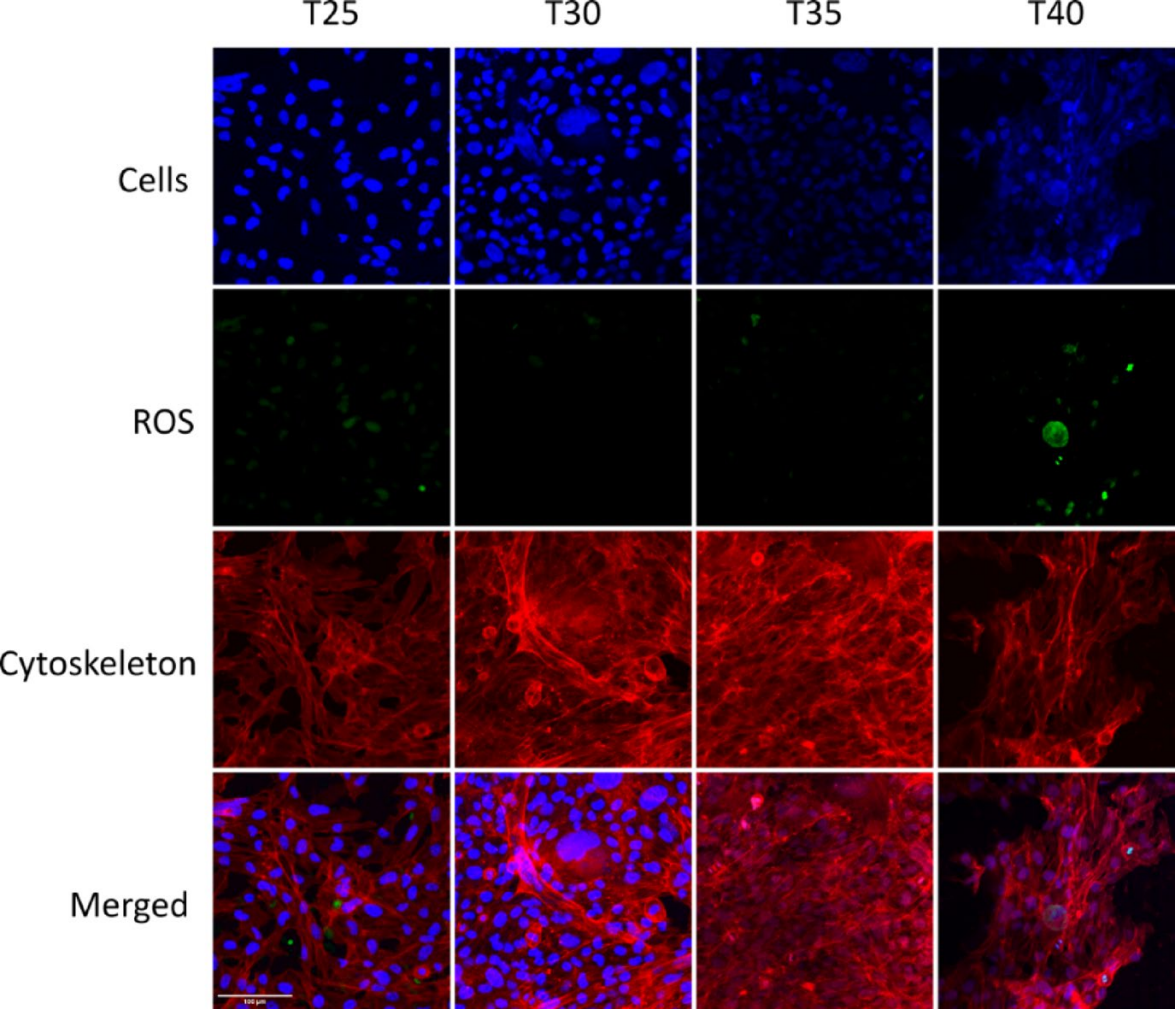
ROS generation assessment after a 24-hour exposure to varying temperature groups on the cell line, hFOB. Representative fluorescent images (magnification: 20x) captured after treatment.

**Fig. 7:**
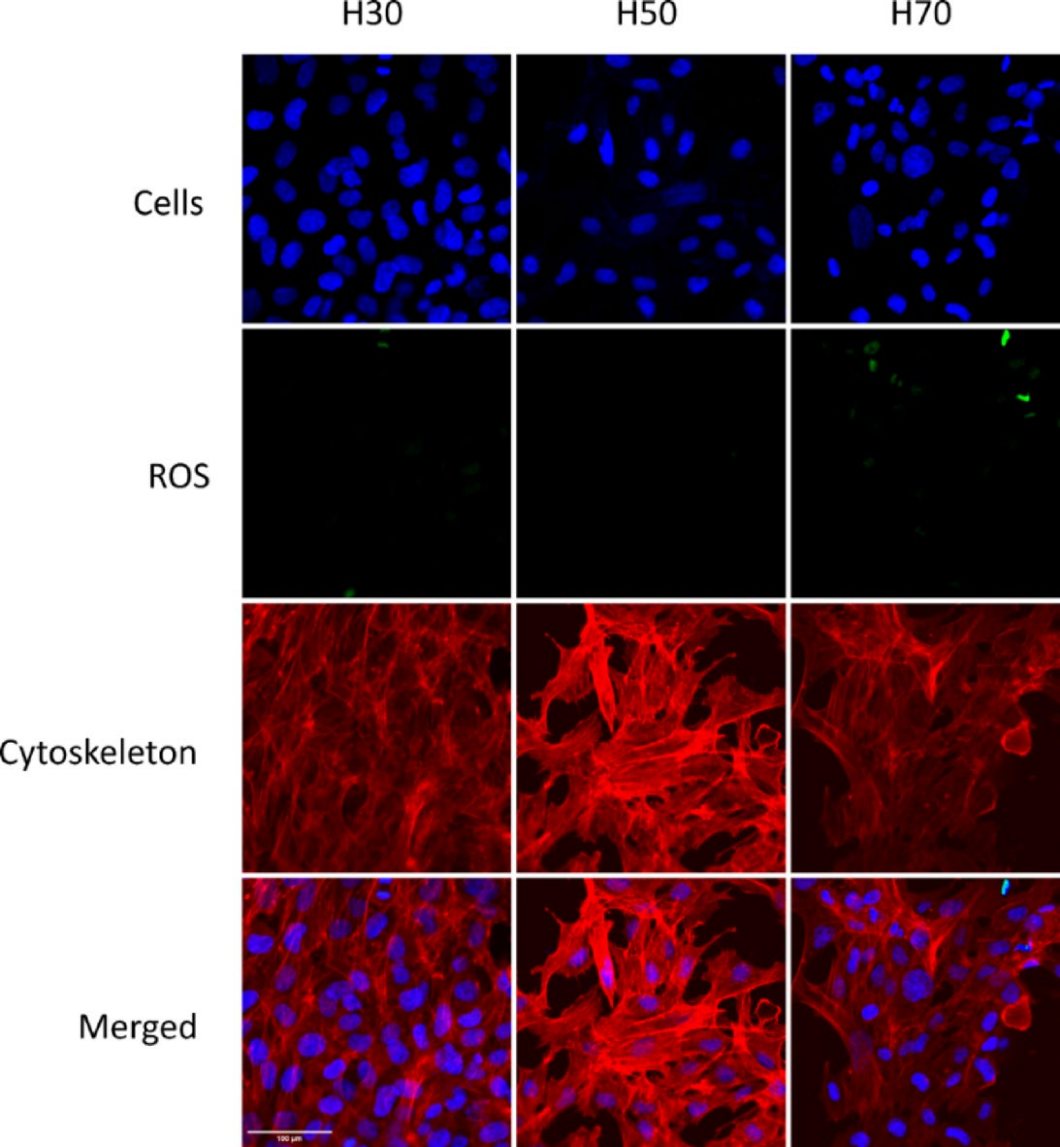
ROS generation assessment after a 24-hour exposure to varying humidity treatment groups on the cell line, hFOB. Representative fluorescent images (magnification: 20x) captured after treatment.

Qualitative analysis of ROS production was used to measure oxidative responses to cell-scaffold interactions after 24 h of exposure. The representative fluorescence images in Figs. 6 and 7 represent nuclear staining in blue (DAPI), intracellular ROS generation as the green fluorescence, and actin cytoskeleton (rhodamine phalloidin) in red, for temperature and humidity scaffolds.

The merged Fig. 6 for the temperature case showed that the observed ROS signal was mostly intracellular, consistent with ROS production in the mitochondria and cytosol. At lower scaffold fabrication temperatures (25–30 °C), ROS levels remained negligible to minimal, as evidenced by weak fluorescence intensity (Fig. 6b). Increasing the temperature increased the number of ROS-expressing cells. Notably, scaffolds fabricated at 40 °C showed an increase in ROS fluorescence intensity, confirming a higher population of ROS-positive cells relative to lower-temperature conditions. This elevation, ROS signals remained localized rather than diffuse, and no deterioration in nuclear morphology or actin cytoskeletal organization was observed.

In contrast, variation in ambient humidity over the range of 30–70% RH did not produce a qualitative change in ROS intensity, with fluorescence remaining weak across all conditions (Fig. 7 merged). However, 70RH showed more cells with ROS activity. Both temperature and humidity variations, nuclei exhibited uniform DAPI staining without evidence of chromatin condensation or fragmentation, and the actin cytoskeleton retained a well-defined structure. Collectively, these findings demonstrate that intracellular ROS generation is sensitive to elevated fabrication temperature and can cause long-term effects at the cellular level. However, considering the role of chronic inflammation and tissue integration impairment caused by sustained ROS, temperature-directed oxidative reactions can have consequences on the scaffold’s ultimate performance and could be investigated further under conditions of prolonged culture or implantation^28,29^.

### Summary and limitations

The investigation of both temperature and humidity during the 3D printing of PLA scaffolds indicates that printing at higher ambient temperatures improves Young’s modulus, and the best biocompatibility for hFOB cells is achieved when PLA scaffolds are fabricated at moderate temperatures (25–35 °C) and low-to-moderate humidity levels (30% RH). The low viability across various settings underscores PLA’s durability as a scaffold material while suggesting that environmental printing factors will influence biological responses. For tissue engineering applications, especially in scaffold fabrication in clinical settings, controlling the printing microenvironment is essential to ensure repeatability and high process control. The observed variations, however slight, may attain significance in applications involving primary cells, sensitive cell types, or bioactive coatings. Ambient temperature and humidity should be further explored to determine the optimal print environment.

#### On cell viability

The highest viability was observed at T35 (~ 91%), suggesting that a slight increase in printing temperatures has no adverse effect on scaffold cytocompatibility. Parallel observations have been documented in research indicating that modest heat fluctuations during PLA printing exert negligible impacts on cell compatibility, although they affect crystallinity and surface roughness, hence altering protein adsorption and cellular growth or adhesion^30^. At 40 °C, a slight decrease to around 86% was observed, possibly suggesting minor thermally induced variations in the surface morphology or chemistry of PLA during the printing process. Higher ambient temperatures during FDM printing have been shown to promote polymer chain rearrangements and alter surface hydrophilicity, possibly affecting cell-material interactions^31^. Excessive humidity during printing might change PLA cooling kinetics, microstructural porosity, and residual moisture content, potentially altering surface wettability and influencing protein adsorption^32,33^. Moreover, elevated ambient humidity could accelerate hydrolytic surface degradation during printing, resulting in localized acidic by-products that hinder cell proliferation and might affect metabolic function^32^.

#### Limitations

This study could be advanced by exploring temperature-humidity combinations, improving trabecular bone geometry, and testing a broader range of materials. In this study, temperature and humidity were tested individually, therefore no interactions between the two factors were observed. Exploring humidity and temperature combinations will allow the determination of optimal printing conditions, accounting for the effects of temperature and humidity on each other. Altering pore size and pore density to better match the architecture of trabecular bone in the proximal femur would improve the accuracy of Young’s Modulus results, increase bone growth, improve osseointegration, and osteoblast proliferation. Trabecular bone has porosity ranging from 50% to 90% and pore sizes ranging from 300 μm to 500 µm^34^. Currently, the average pore size is 660 μm with a porosity of 40%. While the mechanical tests and water capacity tests indicate the repeatability of the designed structure, micro-CT imaging can provide a direct visualization of the internal structure for geometric accuracy, pore size, and porosity, which can be utilized in future work. Testing a variety of materials to assess biocompatibility and strength would also help determine which materials are most viable for tissue regeneration. Several natural and synthetic polymers are currently being explored for bone tissue regeneration, including popular options such as poly (lactic-co-glycolic acid) (PLGA), polycaprolactone (PCL), and polyethylene glycol (PEG), which offer enhanced biocompatibility and biodegradation^35^.

### Conclusions

This study explores the effects of ambient temperature and humidity on FDM print quality. The results indicate that higher ambient temperature increases Young’s modulus, but the biocompatibility for hFOB cells was highest when PLA scaffolds were fabricated at moderate temperatures (25–35 °C) and low-to-moderate humidity (30% RH). Ensuring optimal conditions improves print quality and decreases variability during fabrication. The above is especially important when designing personalized tissue scaffolds, as minor variations may result in increased cell death, reduced cell adhesion, and improper fit, leading to implant failure.

The investigation of the effects of both temperature and humidity on the 3D printing of PLA scaffolds indicates that printing at higher ambient temperatures improves Young’s modulus. The small variability across settings underscores PLA’s durability as a scaffold material while suggesting that environmental printing factors influence biological responses. For tissue engineering applications, especially in scaffold fabrication in clinical settings, controlling the printing microenvironment is essential to ensure repeatability and high process control. The observed variations, however slight, may attain significance in applications involving primary cells, sensitive cell types, or bioactive coatings. Ambient temperature and humidity should be further explored to determine the optimal print environment.

## Methods

### Geometry design

The model for the porous bone scaffold was created in open-source software Blender and then modified in SolidWorks (Dassault Systèmes). In Blender, a random porous structure was generated by applying a Voronoi texture to a cylindrical mesh with a diameter of 2.5 cm and a height of 10.3 cm. These dimensions were chosen to mimic the size of the femoral diaphysis best while still allowing for 3D prints that could be characterized^35^. The pore size was not pre-defined but was calculated from the generated geometry. Trial and error can be used to manipulate pore size to achieve a targeted geometry, if needed in the future.

**Fig. 8:**
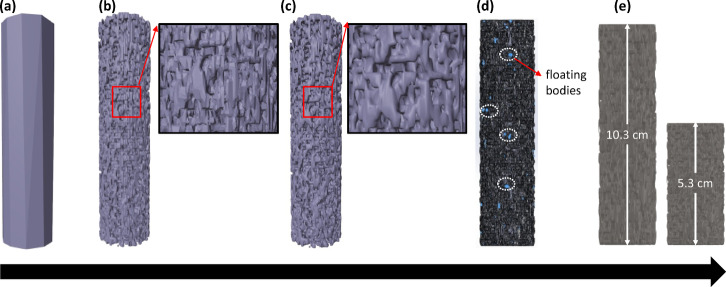
Process of geometry design carried out on Blender and SolidWorks, which includes these steps: **(a)** solid cylinder 1.25 cm x 10.3 cm. **(b)** addition of Voronoi texture, **(c)** shading for smoothness, **(d)** removal of floating bodies, and **(e)** duplication and cut for 5.3 cm scaffold.

The generated rough texture was then manipulated to achieve smooth even surfaces throughout the whole volume. The geometry was imported into SolidWorks as an STL file. In SolidWorks, the disconnected or “floating” bodies were removed, and the geometry was duplicated to generate a scaffold of two heights: 10.3 cm and 5.3 cm. The geometry design method is shown in Fig. 8.

### Printing under controlled temperature and humidity

The scaffold STL file was then imported into AnkerMake slicer for printing. The default print settings were modified by increasing the nozzle temperature to 240 °C and the print bed temperature to 68 °C to improve the flow rate and adhesion. Further changes included reducing the outer brim to 3 mm and eliminating the inner brim. These parameters were established by multiple trial and error to get prints with improved inter layer bonding and print bed adhesion.

**Fig. 9:**
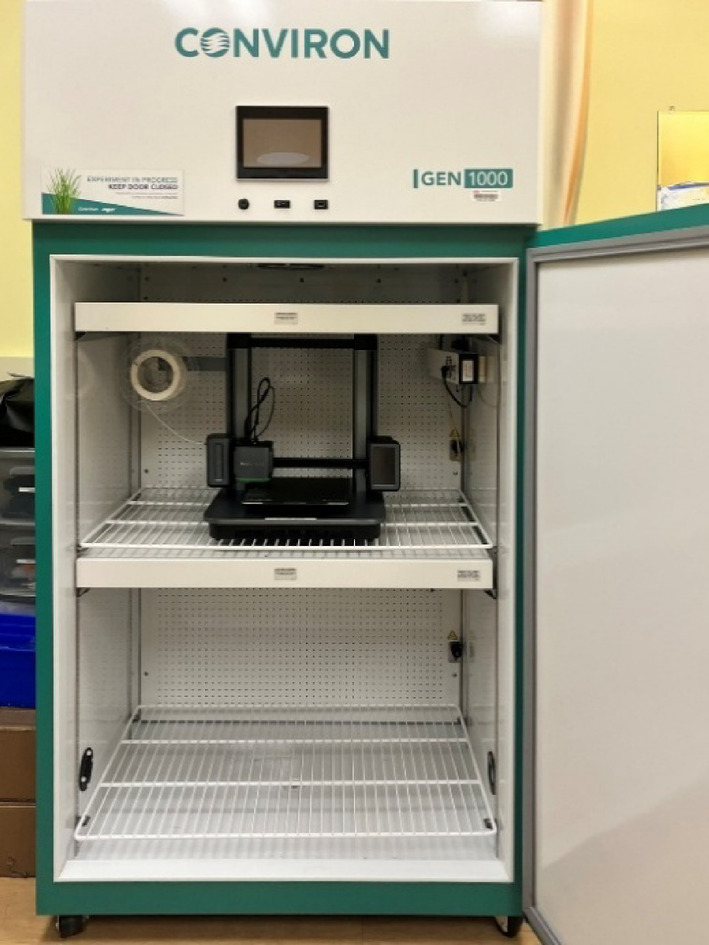
eufyMake M5 FDM printer setup inside CONVIRON Gen 1000 environmental chamber. Midea Cube Dehumidifier is not shown.

The printer was placed in a CONVIRON Gen1000 environmental chamber with controlled temperature and humidity, as shown in Fig. 9. One set of scaffolds was printed at room temperature (recorded as 23.3 ± 0.98 °C) and humidity (48 ± 3%RH), which is considered the “room environment” scaffold. Scaffolds termed as “temperature scaffolds” were printed at controlled temperatures of 25 °C (T25), 30 °C (T30), 35 °C (T35), and 40 °C (T40), all at room humidity (48 ± 3%RH). Additional scaffolds termed “humidity scaffolds” were printed at controlled RH of 30% (RH30), 50% (RH50), and 70% (RH70), all at a fixed temperature of 25 °C. The 30RH condition was achieved by a separate dehumidifier (Midea Cube) placed inside the environmental chamber.

The temperature and humidity were monitored by the chamber as well as an external sensor to ensure accuracy. The desired temperature and humidity were held and monitored for an hour to further verify and stabilize the environment before starting the prints. Three sets of scaffolds at the two lengths of 5.3 cm and 10.3 cm were printed at each selected temperature and humidity, resulting in a total of 48 scaffolds.

### Print geometry characterization

#### Pore size and pore density

**Fig. 10:**
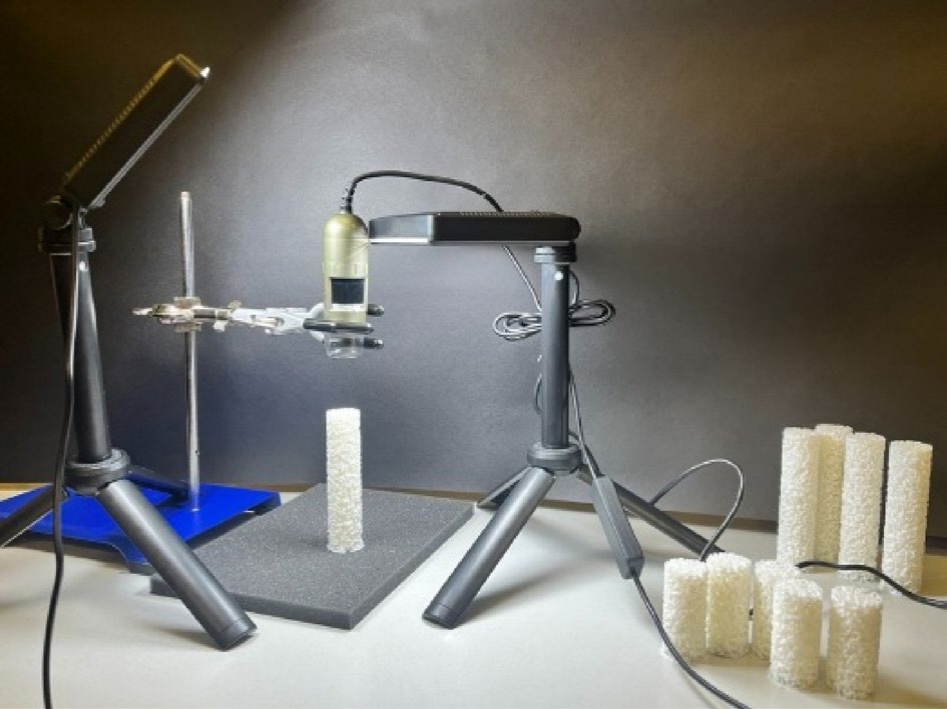
DinoLite imaging setup with porous scaffold samples.

The printed scaffolds were imaged using a DinoLite USB microscope. Figure 10 shows the DinoLite setup used to capture scaffold images. An image of the top face, a magnified section of the top face, and the same magnified section of the top face inverted and grayscaled were taken for each scaffold. An iPhone 13 was utilized to take side images. These images were processed in ImageJ^36^. The magnified, inverted, grayscale images and the image scales were used to determine pore size and pore density using a custom MATLAB script, in which pores were fitted as circles with diameters matching the maximum pore width. Such measurements suffer from an inherent limitation that pores of nonuniform shape require approximations when fitted to a circular shape. Alternative methods, such as local thickness measurement can also be used with ImageJ plugins^37,38^, but circular pore size approximation was chosen here as these values can be related to bone porosity reported in the literature but alternative methods can also be used^39,40^.

#### Water holding capacity

All 5.3 cm scaffolds were weighed when dry, then placed in water and weighed again when wet. The scaffolds were then wrapped and allowed to dry for 24 h. These steps were done three times. Water capacity was then calculated using the equation below.

1 $$\:Water\:Capacity=(Wet\:Weight-Dry\:Weight)/\left(Wet\:Weight\right)$$

### Youngs modulus characterization

The diameter and height of all scaffolds were measured and recorded with a Mitutoyo caliper. After imaging, all 5.3 cm scaffolds were tested with the UnivertS (CellScale Inc) to determine Young’s Modulus through uniaxial compression. The 5.3 cm scaffold was used due to its geometry matching the standard guidelines 2:1 length-to-diameter ratio recommendation to avoid sample buckling as per ASTM D695^41^. The scaffolds were compressed with a metal plate utilizing a force-controlled ramp setting with a max load of 195 N. The selected loading insured strain level was related to psychological strain in the femoral bone, including at high strain levels^42–44^. A total of three consecutive compressions were done with a compression duration of 10 s, a recovery duration of 10 s, and a data collection frequency of 100 Hz. The Young’s Modulus was calculated in Excel.

### Biocompatibility analysis

To determine the biocompatibility (XTT and ROS) of the printed PLA, biocompatibility analysis was performed on broken-down scaffold pieces, which were sterilized before analysis as described below.

#### Sterilization

The 10 cm scaffolds were broken down and weighed to precisely 0.125 g to ensure a constant surface area-to-mass ratio. These scaffolds were sterilized by ultraviolet (UV) irradiation for 30 min to eradicate microbiological contamination without thermally modifying the material. Here, UV was used since the scaffold was broken down for sterilization and biocompatibility analysis. In the alternative case where a full scaffold is used, methods such as ethylene oxide or electron-beam radiation should be chosen to allow sterilization of internal surfaces.

#### XTT assay

Human osteoblast (hFOB) cells were cultivated in growth media (DMEM) under normal conditions (37 °C, 5% CO₂, 95% relative humidity). Cells were inoculated onto 96-well plates at a density of 5,000 cells per well in 250 µL of medium. Sterilized PLA scaffolds were thereafter placed into each well to ensure direct contact between cells and the scaffold. The study was conducted for 48 h to facilitate scaffold–cell contact and potential release of soluble degradation products. Cell viability was evaluated by the MTT/XTT test, which quantifies metabolic activity as an indirect indicator of viable cells. The XTT assay was selected because it produces a water-soluble formazan product, which is directly quantifiable spectrophotometrically without a crystal solubilization step, thereby eliminating variability in porous, leachable-containing constructs during handling. The absorbance was measured at the primary wavelength of the assay (in the present case, 570 nm) to give percent viability normalized to the untreated, unincubated cell control. Cell viability was calculated as below.

2 $$\:\mathrm{Cell\:viability\:(\%)}=\frac{{\mathrm{Absorbance}}_{\mathrm{treated}}}{{\mathrm{Absorbance}}_{\mathrm{untreated}}}\times\:100$$

#### ROS evaluation

Human fetal osteoblast (hFOB) cells were cultured in DMEM enriched with 10% fetal bovine serum and 1% penicillin-streptomycin at 34 °C in a 5% CO₂. Scaffolds were sterilized as before and placed in six-well tissue culture plates containing subconfluent hFOB cells (~ 70%) and incubated for 24 h to enable cell–scaffold interaction. Intracellular reactive oxygen species (ROS) generation was evaluated using a ROS-sensitive cell-permeant ROS fluorogenic probe, 2 7 -dihydrofluorescein diacetate (H2DCFDA). This compound is intracellularly de-esterified and oxidized to a green-fluorescent product with the presence of ROS.

For ROS staining, the cells were treated with a working solution of H2DCFDA - normally 10 µM in serum-free DMEM at 34–37 °C and kept in the dark for 45 min. The dye solution was then aspirated, and the cells were washed twice with PBS to reduce extracellular background fluorescence. To conduct structural co-staining, the cells were fixed with 4% paraformaldehyde in PBS for 10 min at ambient temperature, then washed with PBS and permeabilized using 0.1% Triton X-100 for 5 min at ambient temperature. The actin cytoskeleton was stained using fluorescent phalloidin (red channel, according to the instructions of the manufacturer, generally 20 to 30 min in the dark), and the nuclei were stained with DAPI (blue channel; 5 to 10 min). Following the last PBS washes, fluorescence images were recorded with the help of a Zeiss Axio Observer microscope with a 20x objective, using the following sets of (excitation/emission filters: 405/450nm for DAPI, 488/515nm for ROS, and 561/610nm for phalloidin/Alexa for cytoskeleton). In each of the experimental conditions, one would take a sequence of images of randomly selected fields with the same exposure parameters, therefore, allowing a qualitative comparison of the conditions.

#### Statistical analysis

All experiments were conducted in triplicate (*n* = 3), and all values are represented as mean ± standard deviation. All calculations were performed in Microsoft Excel.

## Data Availability

We have generated data such as imaging and from material testing. All the data is included in the manuscript as figures and images.
